# Single Shot Corrective CNN for Anatomically Correct 3D Hand Pose Estimation

**DOI:** 10.3389/frai.2022.759255

**Published:** 2022-02-21

**Authors:** Joseph H. R. Isaac, Muniyandi Manivannan, Balaraman Ravindran

**Affiliations:** ^1^Department of Computer Science and Engineering, Indian Institute of Technology Madras, Chennai, India; ^2^Touch Lab, Department of Applied Mechanics, Indian Institute of Technology Madras, Chennai, India; ^3^Robert Bosch Center for Data Science and Artificial Intelligence (RBC-DSAI), Department of Computer Science and Engineering, Indian Institute of Technology Madras, Chennai, India

**Keywords:** 3D hand pose estimation, biomechanical constraints, anatomically correct tracking, single shot corrective CNN, depth based hand tracking

## Abstract

Hand pose estimation in 3D from depth images is a highly complex task. Current state-of-the-art 3D hand pose estimators focus only on the accuracy of the model as measured by how closely it matches the ground truth hand pose but overlook the resulting hand pose's anatomical correctness. In this paper, we present the Single Shot Corrective CNN (SSC-CNN) to tackle the problem of enforcing anatomical correctness at the architecture level. In contrast to previous works which use post-facto pose filters, SSC-CNN predicts the hand pose that conforms to the human hand's biomechanical bounds and rules in a single forward pass. The model was trained and tested on the HANDS2017 and MSRA datasets. Experiments show that our proposed model shows comparable accuracy to the state-of-the-art models as measured by the ground truth pose. However, the previous methods have high anatomical errors, whereas our model is free from such errors. Experiments show that our proposed model shows zero anatomical errors along with comparable accuracy to the state-of-the-art models as measured by the ground truth pose. The previous methods have high anatomical errors, whereas our model is free from such errors. Surprisingly even the ground truth provided in the existing datasets suffers from anatomical errors, and therefore Anatomical Error Free (AEF) versions of the datasets, namely AEF-HANDS2017 and AEF-MSRA, were created.

## 1. Introduction

Hand pose estimation in 3D is the task of predicting the pose of the hand in 3D space provided the depth (or 2D) image of the hand. It is used in many fields such as human-computer interactions (Naik et al., 2006; Yeo et al., 2015; Lyubanenko et al., 2017), gesture recognition (Fang et al., 2007), Virtual Reality (VR), and Augmented Reality (AR) (Cameron et al., 2011; Lee et al., 2015, 2019; Ferche et al., 2016). With the advent of deep learning in computer vision, commercial systems such as Oculus™ and LeapMotion™ are shifting from marker-based tracking methods to purely vision-based hand tracking. The shift obliviates the need to wear cumbersome equipment, which affects the user experience. However, marker-less pose estimation is a challenging task as there are several factors such as the complexity of the hand poses, background noise, and occlusions.

**Figure 1:**
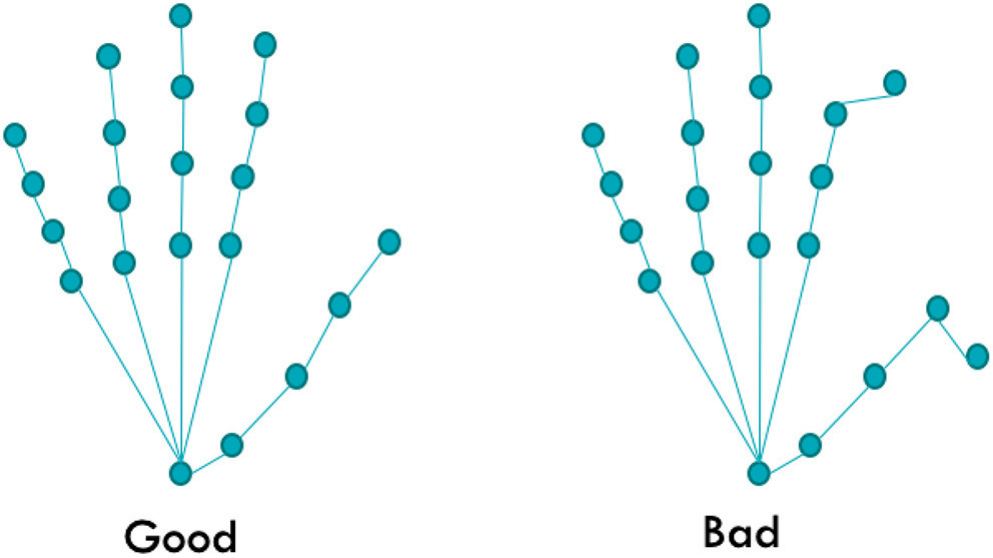
Example of an anatomically incorrect pose. Although most of the joints match the original pose, since two joints are in abnormal angles, the whole pose is considered implausible.

A key problem overlooked by several state-of-the-art models is the realism of the output hand pose. Current state-of-the-art models focus on the accuracy as per the closeness to the ground truth pose of the model rather than the overall anatomical correctness of the model and report low errors in benchmark tests such as the ICVL (Tang et al., 2016), NYU (Tompson et al., 2014), MSRA (Sun et al., 2015), BigHand2.2M (Yuan et al., 2017) and HANDS2017 (Yuan et al., 2017). It is possible to train a model to match almost all hand joints when tested; however, when the error is caused by a finger bent in the opposite direction (as shown in Figure 1), it can affect the user experience. The error can also affect the human system, leading to false information and mismatch in the motor cortex and the visual system (Pelphrey et al., 2005). Hence, in this work, we focus on improving the realism of the predicted hand pose and the validity of the dataset. The main metric used for comparison in this paper is the anatomical error of the hand pose, which is computed by using the joint angles measured for each joint in the hand pose after prediction. These joint angles were compared with the true biomechanical bounds of the hand (discussed in Section 3.1.1). The absolute error between the true bound and predicted joint angle was then calculated for every joint and added together. This value is denoted as the **anatomical error**, and its unit is in degrees. The mean anatomical error of the joints was also reported, and this process was repeated for every hand pose prediction. During the experiments, we observe that the ground truth of the dataset itself contains many anatomical errors in many instances. We address this issue by proposing a new corrected ground truth that conforms to the anatomical bounds of a true human hand.

Earlier approaches to incorporate anatomical information usually take the form of a hand pose filter applied post-facto after the prediction of the pose to correct for anatomical errors (Chen Chen et al., 2013; Tompson et al., 2014; Aristidou, 2018). Post-processing often leads to significant computational overhead. We present a novel approach that we call the Single Shot Corrective CNN (SSC-CNN) that provides a highly accurate hand pose estimation with no anatomical errors by applying corrective functions in the forward pass of the neural network using three separate networks. The term “Single Shot” implies that the model will process the hand-pose and ensure the anatomical correctness in a single forward pass of the network. This ensures that the initial prediction from the network is free of anatomical errors and prevents the need for any correction using a post-processing function.

In summary, our paper provides the following contributions: (1) A novel approach by incorporating biomechanical filter functions in the model architecture, (2) a hand pose estimator that guarantees zero anatomical error while maintaining low deviation from the ground truth pose, (3) we show that these anatomical rules and bounds were not maintained when creating the HANDS2017 and the MSRA hand datasets, and (4) an Anatomical Error Free (AEF) version of the datasets called AEF-HANDS2017 and AEF-MSRA was created. In Section 2 we discuss recent hand pose estimation methods. The proposed architecture is described in Section 3 along with the biomechanical constraints. The experiments to compare the proposed model with the state-of-the-art pose estimators are described in Section 4, and its results are shown in Section 5. We conclude the paper along with our future works in Section 6.

## 2. Related Works

This section discusses hand pose estimation methods that use deep learning algorithms and hand pose estimators with biomechanics-related features such as anatomical bounds.

### 2.1. Pose Estimation With Deep Learning

Hand pose estimation using deep learning algorithms can be classified into discriminative and model-based methods. The former category directly regresses the joint locations of the hand using deep networks such as CNNs (Ge et al., 2017; Guo et al., 2017; Simon et al., 2017; Malik et al., 2018a; Moon et al., 2018; Rad et al., 2018; Cai et al., 2019; Chen et al., 2019; Poier et al., 2019; Xiong et al., 2019). The latter category abstracts a model of the human hand and fits the model with minimum error (such as the mean distance between ground truth and predicted hand pose joints) on the input data (Vollmer et al., 1999; Taylor et al., 2016; Oberweger and Lepetit, 2017; Ge et al., 2018b; Malik et al., 2018b). Directly regressing the joint locations achieves high accuracy poses but suffers from issues such as the hand's structural properties. Works such as Li and Lee (2019) and Xiong et al. (2019) used cost functions taking only the joint locations of the hands into account and no structural properties of the hand. Moon et al. (2018) proposed the V2V Posenet, which converts the 2D depth image into a 3D voxelized grid and then predicts the joint positions of the hand. The cost function of the V2V algorithm used the joint locations alone for training and did not consider biomechanical constraints such as the joint angles.

### 2.2. Pose Estimation With Biomechanical Constraints

Biomechanical constraints are well studied in earlier works to enable anatomically correct hand poses using structural limits of the hands (Ryf and Weymann, 1995; Cobos et al., 2008; Chen Chen et al., 2013; Melax et al., 2013; Sridhar et al., 2013; Xu and Cheng, 2013; Tompson et al., 2014; Poier et al., 2015; Dibra et al., 2017; Aristidou, 2018; Wan et al., 2019; Spurr et al., 2020). Some works such as Cai et al. (2019) used refinement models to adjust the poses with limits and rules. However, most of these works (Ryf and Weymann, 1995; Cobos et al., 2008; Chen Chen et al., 2013; Melax et al., 2013; Sridhar et al., 2013; Xu and Cheng, 2013; Tompson et al., 2014; Aristidou, 2018; Li et al., 2021) apply the rules and bounds after estimating the pose of the hand using post-processing methods such as inverse kinematics and bound penalization. Recent works used biomechanical constraints for hand pose estimation using 2D images in the neural network's cost function to penalize the joints. Malik et al. (2018b) incorporated structural properties of the hand such as the finger lengths and inter-finger joint distances to provide an accurate estimation of the hand pose. The drawback of this method is that the joints' angles are not considered for estimating the pose. Hence the resulting hand pose can still output a pose in which the joint angles can exceed the human joint bounds. Works such as Sun et al. (2017) and Zhou et al. (2017) successfully implemented bone length-based constraints on human pose estimation but only on the whole body and not for the intricate parts of the hand such as finger length constraints. The model designed by Spurr et al. (2020) achieved better accuracy when tested on 2D datasets; however, the model was weakly supervised, and bound constraints were soft. Hence there are poses where the joint angles exceed the anatomical bounds. Li et al. (2021) used a model-based iterative approach by first applying the PoseNet (Choi et al., 2020) and then computing the motion parameters. The drawback of this approach is that it depends on the PoseNet for recovering the primary joint positions and fails to operate if PoseNet fails to predict the pose. Moreover, the resulting search space of the earlier networks still includes implausible hand poses as these models only rely on the training dataset to learn the kinematic rules. We encoded the biomechanical rules as a closed-form expression that does not require any form of training. SSC-CNN's search space is hence much smaller than the aforementioned models. In our approach, the hand joint locations and their respective angles are predicted, and the bounds were implicitly applied to the model such that the joint angle always lies between them. Also, as pointed out in Section 5, many datasets themselves are not free from anatomical errors due to errors during annotation, and hence learning kinematic structures based on the dataset alone might lead to absorbing those errors into our model. To the best of our knowledge, our work is the first to propose incorporating anatomical constraints implicitly in the neural architecture.

## 3. Proposed Framework

This paper's primary goal is to present a framework that provides hand poses that conform to the hand's biomechanical rules and bounds. This goal is achieved by applying the rules implicitly into the forward pass of the neural network. The code for this model is publicly available^1^ and the overall architecture is shown in **Figure 3**.

### 3.1. Biomechanical Structure of the Hand

**Figure 2:**
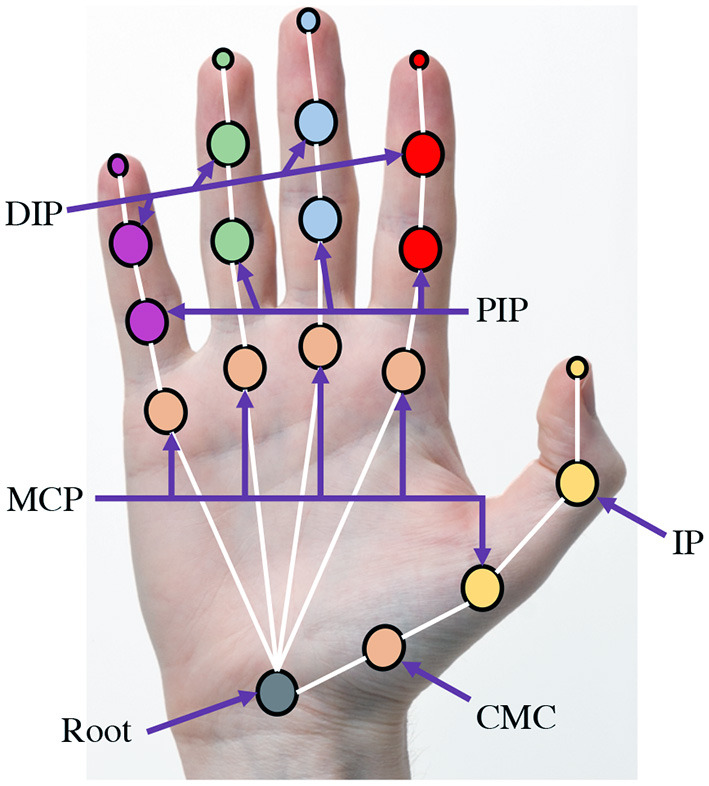
Structure of the human hand. The various joint names are: Metacarpophalangeal (MCP) joint, Distal interphalangeal (DIP) joint, Proximal interphalangeal (PIP) joint, and Carpometacarpal (CMC) joint.

The human hand comprises 27 bones with 39 active muscles, which enable complex tasks such as grasping and pointing (Schwarz and Taylor, 1955; Ross and Lamperti, 2006; Kehr and Graftiaux, 2017). The hand can be simplified to 21 joint locations, each with its degree of freedom and range of motion, and is shown in Figure 2. The fingers of the hand are labeled as the thumb, index, middle, ring, and pinky finger. The key joints for the movements of the hand are (1) Carpometacarpal (CMC) joint, (2) Metacarpophalangeal (MCP) joint, (3) Distal interphalangeal (DIP) joint, and (4) Proximal interphalangeal (PIP) joint.

The wrist joint is the root of the hand and is simplified to 6 degrees of freedom (DoF) as it is the result of the chain of movements from the shoulder to the arm. The CMC joint is connected to the wrist joint, and consists of 3 DoFs as per (Chim, 2017): (1) abduction/adduction, (2) flexion/extension, and (3) rotation. One MCP joint is connected to the CMC joint, while the other 4 MCP joints are connected to the wrist. The thumb MCP joint's function is slightly different from the other MCP joints as the thumb MCP joint has only 1 DoF (flexion/extension), whereas the other MCP joints have 2 DoFs each. The remaining joints are the interphalangeal (IP) joints comprised of two types, namely distal and proximal (DIP and PIP) joints. The IP joints are 1 DoF each for flexion and extension alone. The thumb has one DIP joint and does not have a PIP joint.

#### 3.1.1. Biomechanical Bounds

According to the works of Ross and Lamperti (2006) and Hochschild (2015), the angular bounds of the joints are consolidated and these rules and bounds are all incorporated in the SSC-CNN architecture:

- CMC joint: 45° abduction and 0° adduction, 20° flexion and 45° extension, and 10° of rotation.
- Thumb MCP: flexion 80° and extension 0°. Other 4 MCPS: flexion 90° and extension 40°, as well as abduction 15° and adduction 15°.
- PIP joints: flexion 130° and extension 0°
- DIP joints: flexion 90° and extension 30°

### 3.2. SSC-CNN Architecture

**Figure 3:**
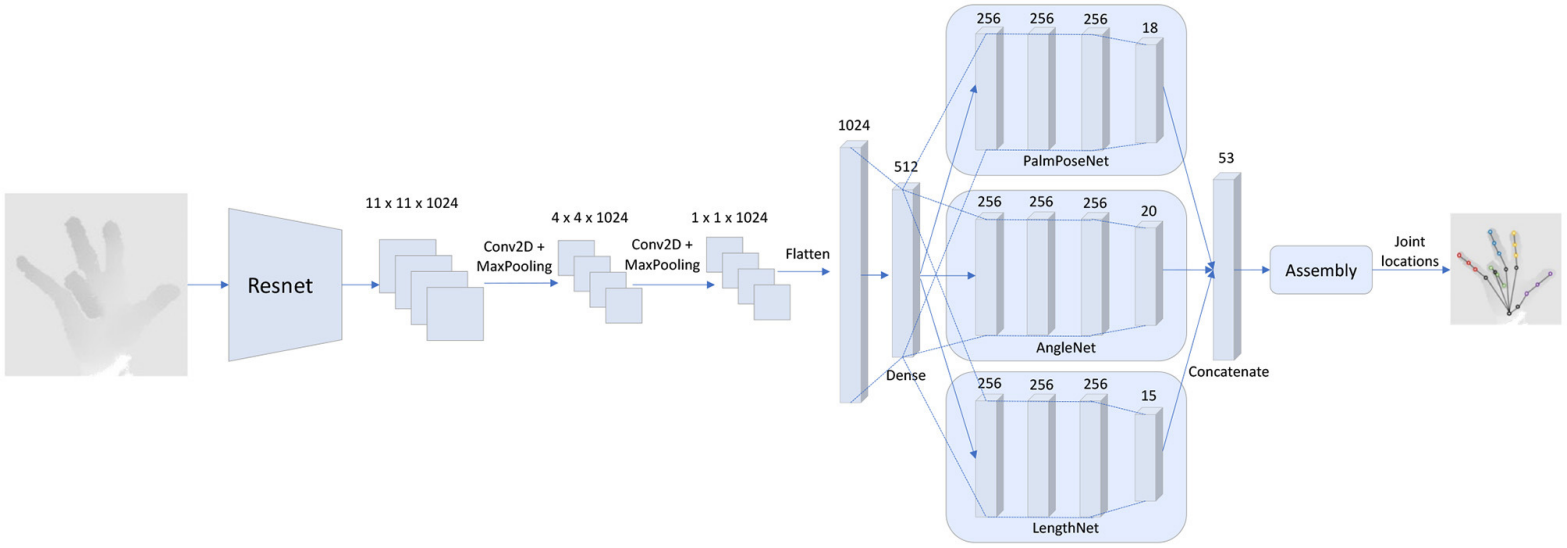
Framework architecture.The proposed architecture uses part of the Resnet50 model for feature extraction. The features then pass through two sets of convolutional layers and max pooling layers and then flattens to a common dense layer. This layer is then fed as input to three sub-nets: (1) The PalmPoseNet, which outputs an 18-dimensional vector corresponding to the 3D positions of the palm joints (root joint, MCPs and CMC), (2) the AngleNet, which outputs a 20-dimensional vector corresponding to the joint angles of the hand and (3) the LengthNet which outputs an 18-dimensional vector corresponding to the length of each finger segment of the hand. The features are then concatenated and sent as input to the assembly which then provides the joint locations as output.

The Resnet50 model (He et al., 2016) is used as a backbone for the SSC-CNN (architecture shown in Figure 3). The layers up to “**conv4_block6_out**” are used (after 6 block computations of the Resnet50), and the weights were transferred from the model trained on the ImageNet dataset (Deng et al., 2009). An input image of size 176 × 176 × 3 is provided to the pre-trained Resnet50 model. The output of this layer is then fed to a convolutional layer (1024 filters of size 3 × 3 with ReLU activation) and then a max-pooling layer (2 × 2). The size of the features at this time is 4 × 4 × 1024 which is sent through another convolutional layer and max-pooling with the same configuration as before and then flattened to a 1024-dimensional vector. The compressed set of features is passed to a single dense layer of size 512 using ReLU activation which is called the *common dense layer*. This is then sent to three individual networks for regressing the hand's various characteristics, which then predicts the pose of the hand using an assembler. The three individual networks are called: (1) PalmPoseNet, (2) AngleNet, and (3) LengthNet.

#### 3.2.1. PalmPoseNet

The PalmPoseNet predicts the joint locations of the root joint, the CMC joint of the thumb, and 4 MCP joints (the thumb MCP is excluded as the root joint is the CMC joint). These joints do not have any strong biomechanical bounds and are dependent on the user's palm-size and structure. Hence to make the model robust, these points are directly regressed by the PalmPoseNet. The 512 features from the common dense layer are taken as input to three dense layers which has 256 nodes each using the sigmoid activation function. The features then pass to a final dense layer with 18 nodes which also uses a sigmoid activation function and these 18 points correspond to the 3D location of the six joints.

#### 3.2.2. AngleNet

The AngleNet provides the angle of each joint of the fingers. As there are five fingers, including the thumb, and each finger has four angles associated with it (as explained in Section 3.1), there are a total of 20 angles that are regressed by the AngleNet. The 512 features from the common dense layer are taken as input to three dense layers which has 256 nodes each using the sigmoid activation function. The features then pass to a final dense layer with 20 nodes that uses a sigmoid activation function. These 20 features are then used in the composition of the hand pose as described in Section 3.3.

#### 3.2.3. LengthNet

The LengthNet provides the length of the individual segments of the fingers of the hand, such as the length of the part between the thumb CMC to the thumb MCP and the thumb MCP to thumb IP. The 512 features from the common dense layer are taken as input to three dense layers which has 256 nodes each using the sigmoid activation function. The features then pass to a dense layer with 15 nodes that uses a sigmoid activation function. These 15 features are relative values to calculate the segments' lengths which are then used in the composition of the hand pose as described in Section 3.3.

### 3.3. Assembly of the Pose

**Figure 4:**
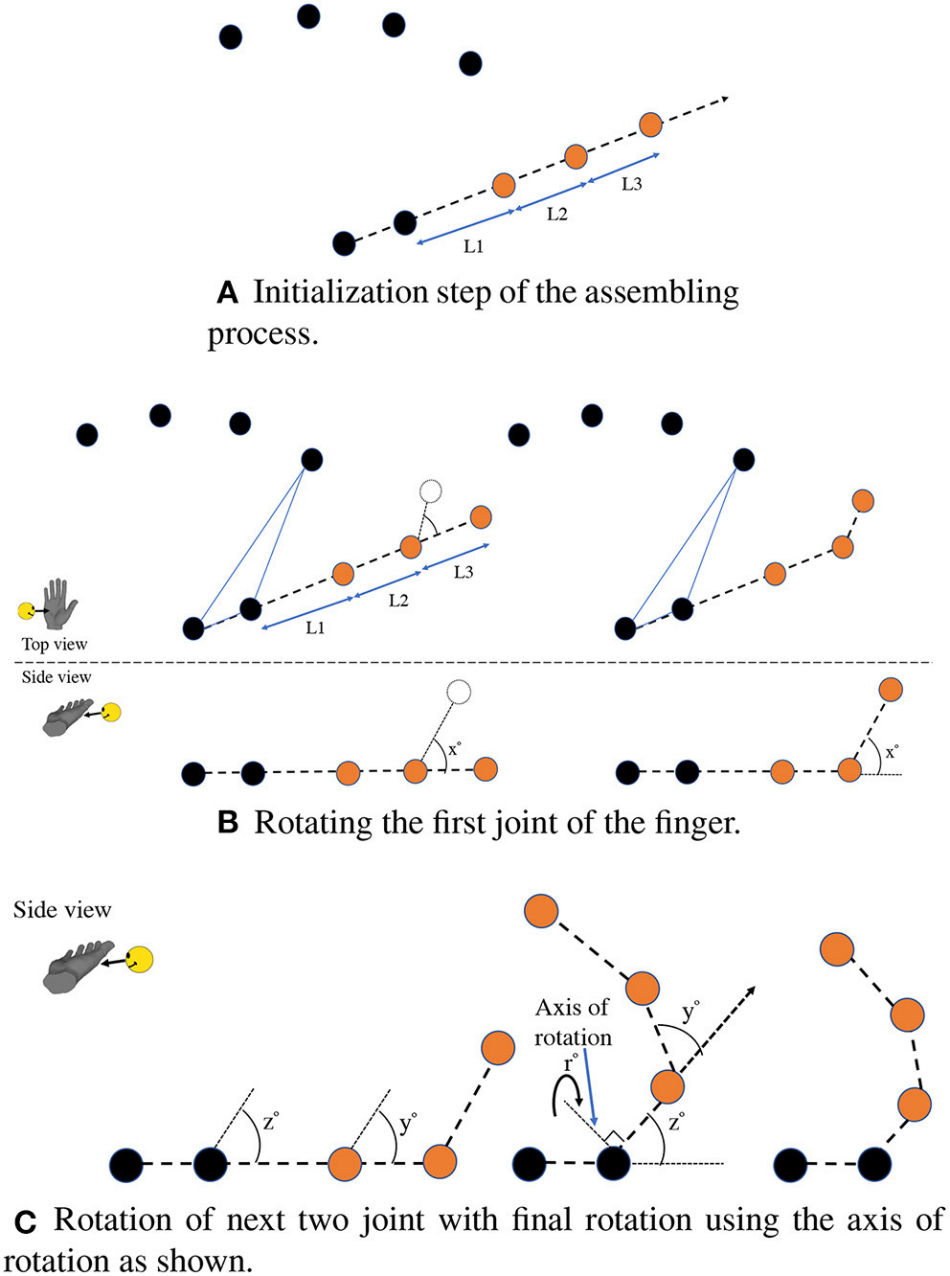
Overall sample process of the assembly to create a thumb of the hand pose. The initialization step shown in **(A)** uses the 3D positions of the joints directly regressed from the PalmPoseNet and then uses the root joint with the CMC joint to extrapolate a 3D line. The three joints are then placed on this line, and the length of each segment between the joints is taken from the LengthNet. The tip joint is first rotated using the axis, which passes the adjacent joint and is parallel to the plane created by the root joint, CMC joint, and the index MCP joint. The second and third joints rotate similar to the previous joint as shown in **(B)** using their axes of rotation. The last rotation uses an axis perpendicular to the line that passes the CMC and the current third joint position **(C)**.

The assembly is a non-trainable portion of the architecture responsible for constructing the resulting hand pose based on the values from the previous individual networks. A sample process flow of the assembly for one finger (the thumb) is shown in Figure 4, and this process repeats for each finger.

The first step is to take the 3D positions from the PalmPoseNet and use these points as the reference for the fingers. Taking the thumb as an example sequence, the next step is to use the root joint and the CMC joint as line points and extrapolate the line beyond the CMC joint for placing the thumb joints as shown in Figure 4A. The length of each segment between the joints is taken from the LengthNet.

The LengthNet output vector is from a sigmoid function and hence ranges from 0 to 1. These values are multiplied with a hyper-parameter (γ) which is the longest possible length of a finger segment. Works from Sunil (2004) and Chan Jee and Yun (2016) include studies where the individual parts of the hand are measured. Using this data, we set γ = 80 mm which is the maximum length of an average finger segment as per these studies. Using a sigmoid-based output for the individual lengths provides finer control for the model during training.

After extrapolating the thumb joints, the next step is to rotate the joints to their corresponding angles, as shown in Figure 4B. The values from AngleNet are used for setting the angles of rotation. These values also range from 0 to 1 as the sigmoid activation function is used. Each value is then multiplied according to the biomechanical range of the joint. This ensures that the range of the angle does not overshoot or undershoot the range of the joint and is shown in equation 1.


(1) $$\begin{array}{l} \theta^{i}=\left(A^{i}*\left(\theta_{\text{Upper}}^{i}-\theta_{\text{Lower}}^{i}\right)\right)+\theta_{\text{Lower}}^{i} \end{array}$$

where θ^*i*^ is the *i*-th joint angle, *A* is a vector from the AngleNet, $\theta_{\text{Lower}}^{i}$ is the lower bound of θ^*i*^ and $\theta_{\text{Upper}}^{i}$ is the upper bound of θ^*i*^. For example, the thumb IP ranges from −30° (considering extension as negative) to 80° (flexion as positive) and if *A*^*i*^ = 0.2 then θ^*i*^ = (0.2*(80−(−30)) + (−30) = −8. This value lies in the range [−30, 80].

To rotate the joint by an angle, a reference plane is required. The root joint is always used as one point of the reference plane, while two adjacent MCP joints will be used as the other two points for each finger. The plane used for the thumb rotation is formed by the root joint, CMC joint, and the index MCP joint. Similarly, for the index finger, the index MCP and the middle MCP is used. For the last finger, i.e., the pinky, the pinky MCP and the ring MCP are used, and the rotation signs are inverted.

The finger's tip is the first joint to be rotated, as shown in Figure 4B. To rotate the joint, an axis of rotation must be calculated. This axis is created using a vector from the adjacent joint, which lies on the reference plane and is perpendicular to the line from the first joint to the adjacent joint. After the first joint rotation, the next joint in the chain is rotated using an axis vector constructed in a similar fashion to the first joint and originating from the next adjacent joint. The second joint rotation is also applied on the first joint using the same axis of rotation. The chain then continues for the third joint using the axis originating from the next adjacent joint in the line where the first and second joint also rotates. After the three rotations are performed, the last rotation takes place with an axis originating from the last joint (CMC in case of thumb and MCP for other fingers) and is projected perpendicular to the line from the current joint to the previous joint. The three joints are then rotated around this axis as shown in Figure 4C. This whole process is repeated for each finger, resulting in the overall pose of the hand.

### 3.4. Loss Function of SSC-CNN

As the assembly module is non-trainable, the loss function is calculated using the 53-dimensional vector after the concatenation phase. The assembly process is invertible and hence the joint locations of the ground truth is converted to the target 53-dimensional vector and the loss function is calculated as shown in equation 2.


(2) $$\begin{array}{l} L=\frac{1}{6}\overset{6}{\underset{i=1}{\sum }}∥\hat{P_{i}}-P_{{\text{GT}}_{i}}{∥}^{2}+\frac{1}{15}\overset{15}{\underset{i=1}{\sum }}|\hat{L_{i}}-L_{{\text{GT}}_{i}}|+\frac{1}{20}\overset{20}{\underset{i=1}{\sum }}|\hat{A_{i}}-A_{{\text{GT}}_{i}}| \end{array}$$

where *P* is the vector of joint locations from the PalmPoseNet, *L* is the vector of lengths derived from the LengthNet and A is the vector of angles derived from the AngleNet. *P*_GT_, *L*_GT_, *A*_GT_ are the ground truth vectors which are derived by using the reverse assembly process. The loss *L* consists of three parts, (1) the mean euclidean distance between the ground truth and predicted PalmPoseNet joint locations, (2) the mean absolute difference between the ground truth and predicted lengths and (3) the mean absolute difference between the ground truth and predicted angles. Hence the gradients are computed on the pre-final output that comes before the assembly phase and not on the assembled pose (joint locations) of the hand.

### 3.5. Dataset Used

The proposed framework was tested on two popular datasets, namely the MSRA (Sun et al., 2015) and HANDS2017 (Yuan et al., 2017) datasets. These datasets were used as they use the true joint locations such as the MCP joint and CMC joint locations compared to the edge centers used by the NYU (Tompson et al., 2014) dataset. The MSRA dataset comprises about 70,000 images, and HANDS2017 has more than 900,000 train images and 250,000 test images. The SSC-CNN was trained on these dataset's training sets and tested their respective test sets using the same architecture. No anatomical corrections were made on the datasets' ground truth during training to maintain consistency with other state-of-the-art models during comparison.

## 4. Experiments Performed

As this framework focuses primarily on the anatomical correctness of the pose instead of the supposed accuracy as reported by other papers, we performed comparative tests of anatomical correctness on other state-of-the-art models and datasets along with the accuracy metrics.

### 4.1. Error of Model After External Correction

To study the change in the accuracy of the model when correcting the anatomical error of the model, a corrector module was designed based on our earlier work (Isaac et al., 2021) so that it can take the hand poses of the current state-of-the-art models as input and correct the anatomical errors of the model. This module is plugged into each test model and used to correct the anatomical error, and the correction's strength is adjusted using a factor α.

#### 4.1.1. Corrector Module Construction

The module utilizes the bounds explained in Section 3.1.1 and corrects the pose of the hand according to the bounds. The first step is to calculate the joint angles from the 3D joint locations provided by the estimator. To keep the origin of rotations and measurements consistent between hand poses, the hand pose is temporarily aligned to the XY plane using affine 3D transformations. The code for the corrector module is publicly available^2^.

The second step is to calculate the deviation of each joint from its limit. Considering the current joint angle of a particular joint as θ_c_ = [θ_*x*_, θ_*y*_, θ_*z*_], where θ_*x*_, θ_*y*_, and θ_*z*_ are the individual Euler angles to each axis, the anatomical error of the particular joint is derived in equation 3.


(3) $$\varepsilon_{\text{d}}^{\theta }=\left\{ \begin{array}{l} \begin{array}{cc} \theta_{\text{d}}-\theta_{\text{upper}} & \text{if}\theta_{\text{d}}>\theta_{\text{upper}}\\ \theta_{\text{lower}}-\theta_{\text{d}} & \text{if}\theta_{\text{d}}<\theta_{\text{lower}} \end{array}\\ \begin{array}{cc} 0 & \text{ otherwise} \end{array} \end{array}\right.\text{where }d=x,y,z$$

The third step is to correct the joint's angle using the error derived from equation 3. The correction's strength is adjusted using a factor α and is shown in equation 4.


(4) $$\theta_{\text{d}(\text{new})}=\left\{ \begin{array}{l} \begin{array}{cc} \theta_{\text{d}}-\alpha *\varepsilon_{\text{d}}^{\theta } & \text{if}\theta_{\text{d}}>\theta_{\text{upper}}\\ \theta_{\text{d}}+\alpha *\varepsilon_{\text{d}}^{\theta } & \text{if}\theta_{\text{d}}<\theta_{\text{lower}} \end{array}\\ \begin{array}{cc} \theta_{\text{d}} & \text{ otherwise} \end{array} \end{array}\right.$$

where *d* = *x, y, z* and α ∈ [0, 1]. If α = 0, then there is no correction and the resultant angle is the original angle. If α = 1, then the angle is 100% corrected based on the hand's biomechanical rules.

### 4.2. Ground Truth Validation

To validate the anatomical correctness of the ground truth, the anatomical error of the ground truth labels is calculated, and the ground truth is also compared with itself after external correction using the corrector module described in Section 4.1.1.

## 5. Experiment Results and Discussion

**Figure 5:**
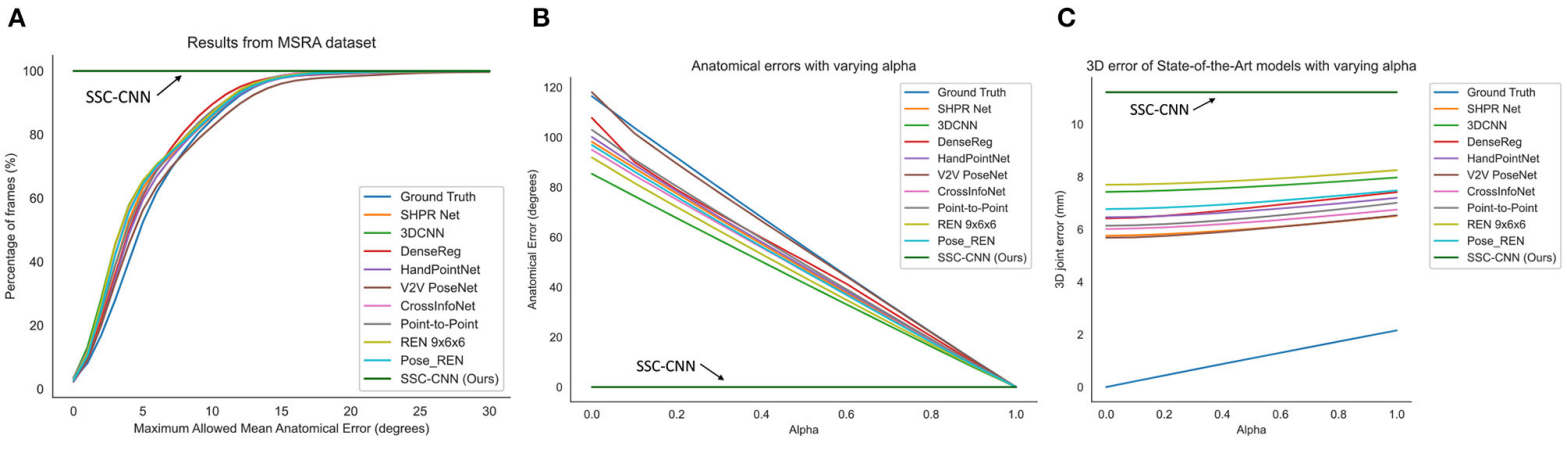
Comparison of the anatomical errors and the 3D joint errors of various state-of-the-art models along with our proposed model using the MSRA hand dataset **(A,C)**. The ground truth is also shown for comparison as it has high anatomical errors **(B)**. The error can be due to the noises during the recording of the lab.

**Figure 6:**
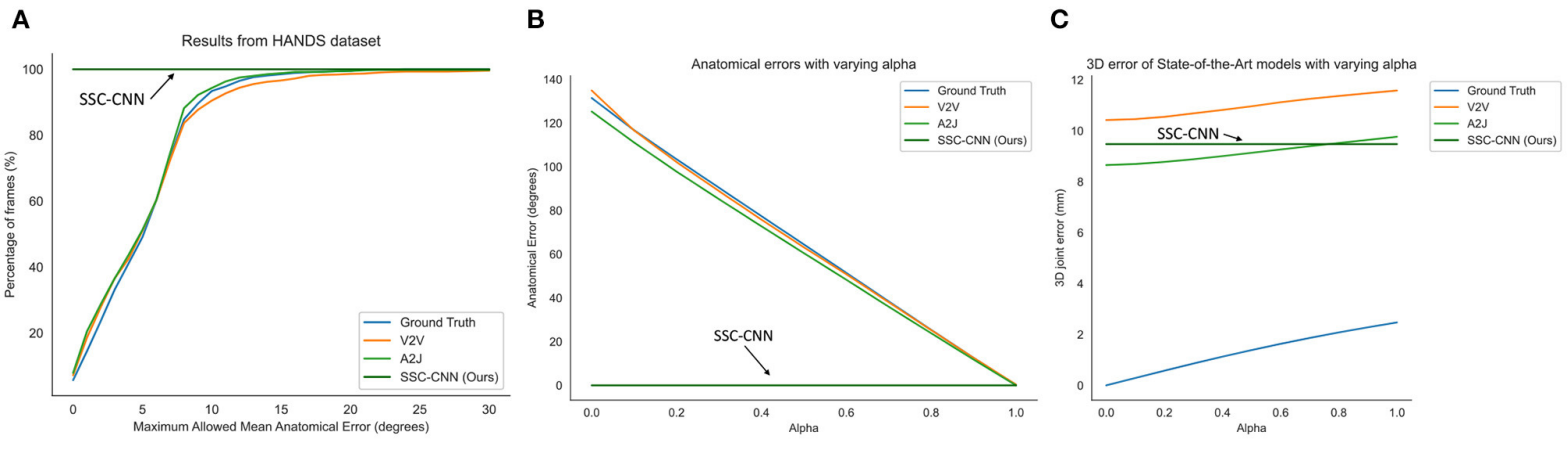
Comparison of the anatomical errors and the 3D joint errors of various state-of-the-art models along with our proposed model using the HANDS hand dataset **(A,C)**. The ground truth is also shown for comparison as it has high anatomical errors **(B)**. This can be due to the noises during the recording of the labels.

**Figure 7:**
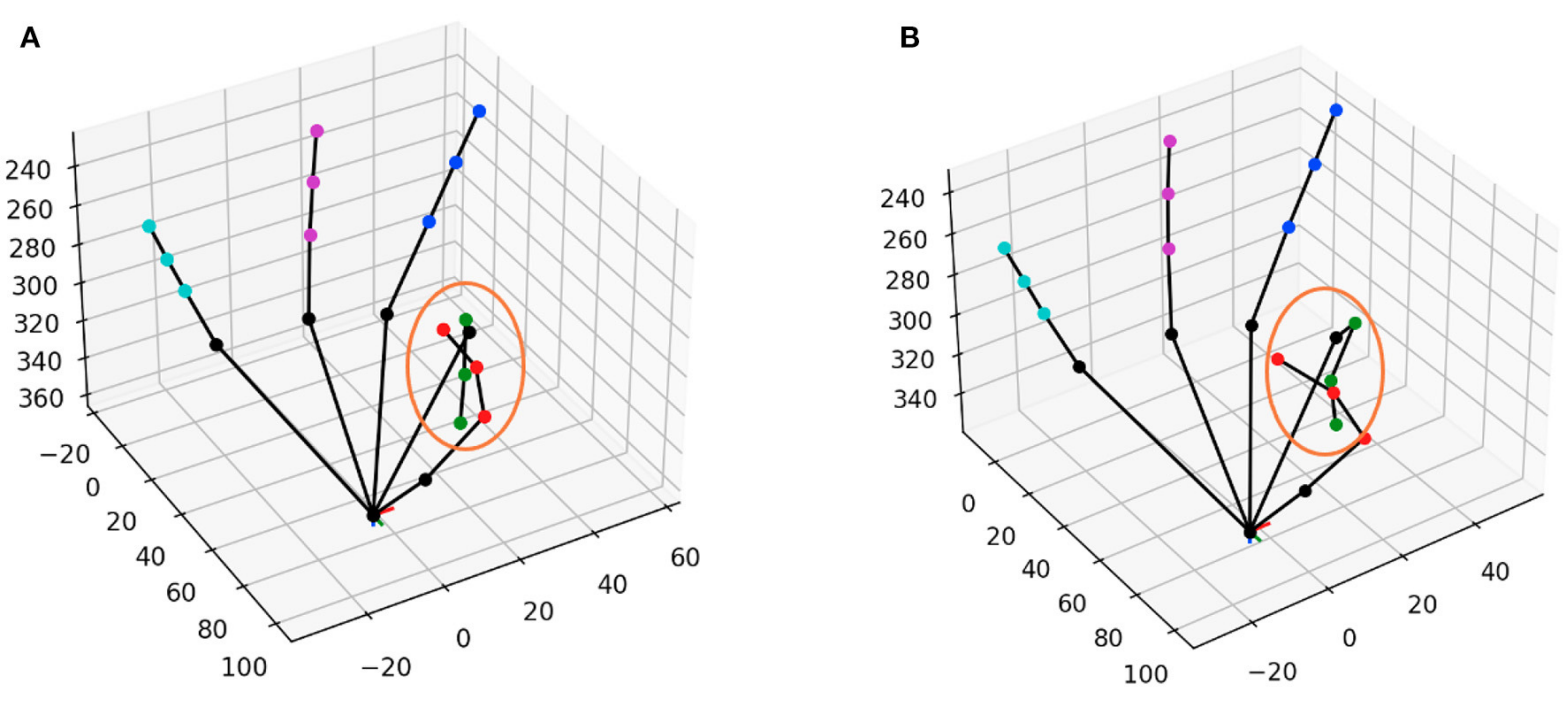
Qualitative comparison between the pose from the MSRA dataset using **(A)** SSC-CNN and **(B)** the same pose from Wang et al. (2018). The circle shows the part which is anatomically wrong in **(B)** while its correctly shown in **(A)**.

Figures 5, 6 shows the comparison of the models using the MSRA hand gesture (Sun et al., 2015) and HANDS2017 (Yuan et al., 2017) datasets, respectively. A qualitative comparison is shown in Figure 7 between a pose from SSC-CNN and another state-of-the-art model. For the first graph of the two sets (Figures 5A, 6A), the x-axis shows the maximum allowed mean anatomical error (calculated as mean per joint per hand), and the y-axis denotes the percentage of frames of the dataset, which is up to the specified mean anatomical error. For context, the steeper the curve is in the graph, the better the model in terms of anatomical correctness. Our model has no anatomical errors and hence the steepest line in both datasets. The second part of the set (Figures 5B, 6B) shows the total anatomical error (calculated as mean per hand and not per joint to show the difference) of the model per hand frame using the correction module set at each value of α at steps of 0.1. The third graph (Figures 5C, 6C) represents the 3D joint error which is the mean Euclidean distance from the predicted joint to the ground truth joint. The ground truth used in the test is not anatomically corrected and is the original ground truth. The lowermost line seen in both graphs is the dataset's ground truth compared with itself after anatomical correction. As seen in the graphs, the ground truth itself has high anatomical errors, and a likely cause of this anatomical discrepancy is the method used in creating the datasets.

**Figure 8:**
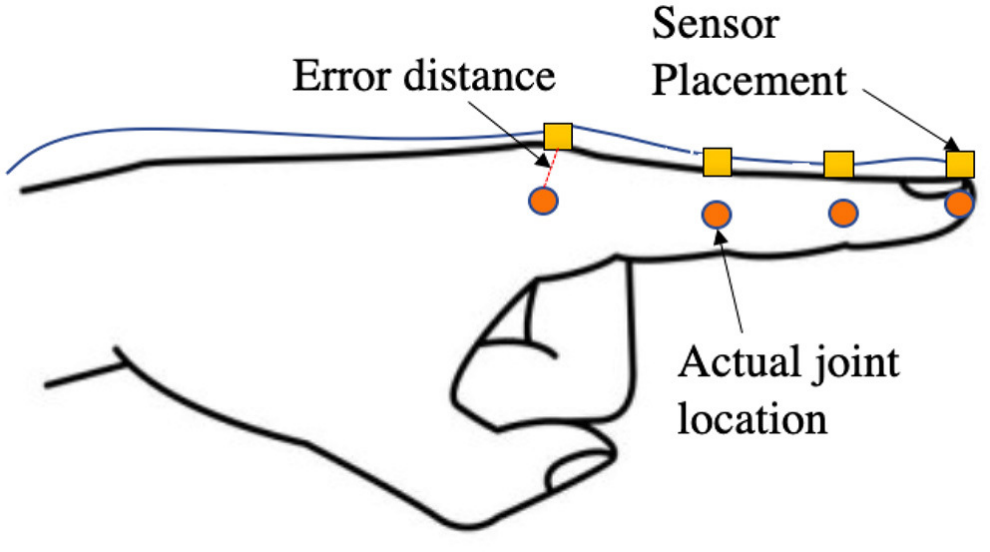
Illustration to show the error in the true position of the hand when measuring using a sensor placed on top of the finger. There is a small gap since the sensor placement is superficial, and the true position of joints that lie inside the hand will have large errors.

As shown in the works of Oberweger et al. (2016), the ground-truth curated in the HANDS and MSRA datasets are not exact representations of the real hand poses. The MSRA dataset uses a combination of the author's hand pose estimator as a reference with manual editing, which is tedious and prone to human errors as seen in Table 1. The HANDS2017 dataset was recorded using the Ascension Trakstar™ ^3^bwhich is reported to have an accuracy of ±1.4 mm and is attached on top of the finger during recording. As shown in Figure 8, if the sensor is placed on top of the finger during the recording of poses, the joint's actual position will be at an offset from the recorded position of the hand. Hence the ground truth may not always be the actual position of the hand for many frames. With anatomically incorrect models, the error to the ground truth (non-corrected) can tend to 0. However, our algorithm puts emphasis on the anatomic correctness over the closeness to the ground truth. Hence this resulted in a relatively higher 3D joint error of 9.48 mm using the HANDS2017 dataset and 11.42 mm using the MSRA dataset as compared to the state-of-the-art models. However, our model shows comparable results when using the correction module as these models have very high anatomical errors, and correcting these errors increases the 3D joint location error. To help the community for future hand tracking related works, we also provide our corrector module publicly available to correct the ground truth of the HANDS2017 and MSRA datasets to create an Anatomical Error-Free (AEF) version of those datasets.

**Table 1:** Anatomical Errors (AE) of the HANDS2017 dataset and MSRA dataset ground truth and the 3DJE of the corrected ground truth to the non-corrected version.

**Dataset**	**AE with no correction (**°**)**	**AE with correction (**°**)**	**3DJE after correction (mm)**
HANDS2017	131	0	2.38
MSRA	116	0	1.89

To study the effect of the sub-networks, ablation studies were performed by removing the subnetworks and regressing all the joints of the hand directly. The resultant hand poses did not conform to the biomechanical rules and had joints rotated by abnormal angles as well as abnormally long finger segments at times. This behavior shows that the subnetworks ensure that the hand pose conforms to the angle bounds and proper finger lengths.

Tables 2, 3 contain the 3D error of the hand pose estimators before and after application of the corrector module. The anatomical error before using the module is also shown in the tables. As seen in the table, our model predicts poses with no anatomical errors and has the same 3DJE as these bounds are implicitly coded in the model's architecture, and the resulting hand poses always conform to these bounds. Whereas other models have large anatomical errors and deviates after correction.

**Table 2:** 3D Joint Errors (3DJE) and Anatomical Errors (AE) derived from 40,000 images from the HANDS2017 dataset with all the models.

**Model**	**3DJE with no correction (mm)**	**AE with no correction (**°**)**	**3DJE with correction (mm)**
**SSC-CNN (Ours)**	9.48	**0**	**9.48**
A2J (Xiong et al., 2019)	**8.65**	125	9.73
V2V Posenet (Moon et al., 2018)	10.42	135	11.27
Ground truth	0	131	2.38

*The first column contains the name of the model, the second column has the errors of the model with no external correction, the third column has the Anatomical Error (AE) of the model without correction and the fourth column has the errors of the model after correction. Bold numbers denote the smallest value present in the column excluding the underlined values which is from the ground truth of the dataset*.

**Table 3:** 3D Joint Errors (3DJE) and Anatomical Errors (AE) derived from 20,000 images from the MSRA dataset with all the models.

**Model**	**3DJE with no correction (mm)**	**AE with no correction (**°**)**	**3DJE with correction (mm)**	**3DJE with correction compared to corrected ground truth (mm)**
**SSC-CNN (Ours)**	11.42	**0**	11.42	11.32
SHPR Net (Chen et al., 2018)	7.86	98	8.56	7.92
3DCNN (Ge et al., 2017)	9.48	85	10.05	9.55
DenseReg (Wan et al., 2018)	7.73	107	**8.37**	7.80
HandPointNet (Ge et al., 2018a)	8.31	100	9.14	8.55
V2V Posenet (Moon et al., 2018)	**7.59**	118	15.37	14.84
CrossInfoNet (Du et al., 2019)	7.96	103	8.41	**7.75**
Point-to-Point (Ge et al., 2018b)	7.71	95	8.51	7.91
REN 9x6x6 (Wang et al., 2018)	9.79	91	10.20	9.66
Pose REN (Chen et al., 2020)	8.65	96	9.19	8.59
Ground truth	0	116	1.89	0

*The first column contains the name of the model, the second column has the errors of the model with no external correction, the third column has the Anatomical Error (AE) of the model without correction, the fourth column has the errors of the model after correction and the last column has the errors of the model when compared to a correction version of the ground truth. Bold numbers denote the smallest value present in the column excluding the underlined values which is from the ground truth of the dataset*.

## 6. Conclusion, Limitations, and Future Works

We proposed a novel framework called the SSC-CNN for 3D hand pose estimation with biomechanical constraints. The network has biomechanical rules and bounds encoded in the architecture level such that the resulting hand poses always lie inside the biomechanical bounds and rules of the human hand, and no post-processing is required to correct the poses. Our framework was compared to several state-of-the-art models with two datasets. Experiments have shown that the SSC-CNN has comparable results but with no anatomical errors, whereas the state-of-the-art models have very high anatomical errors. The ground truth of the datasets also has anatomical errors, and anatomically error-free versions were created.

Our framework has a limitation in which the training phase requires data pre-processing to derive the joint angles as these angles were not available in the datasets used. Another limitation is that our hand pose estimator does not take the velocity of the joint movements into consideration when correcting them. The angular velocity of the joints also has biomechanical constraints, and these will be incorporated in future works for the model. Although the model is highly robust for varying palm sizes, extreme cases like estimating the hand poses of children may result in inaccurate poses as the dataset used for training does not cover young children's hands and can be investigated in a future work.

Future works also include using synthetic datasets such as the MANO hands (Romero et al., 2017) so that the ground truth will be assured of the hands' true location along with children's hand poses. Using these synthetic datasets, we can also compare the spectrum of poses covered by the currently available datasets and hence cover a broader spectrum of poses for training. Analyzing the history of the hands' motion using methods such as recurrent neural networks (Yoo et al., 2020) instead of processing only one instance of the hand can avoid erratic motions during self-occlusions and will be investigated in another study for adding the feature to the SSC-CNN. The history can include the velocity and acceleration of the joint motions, which also have biomechanical bounds and further enhance the pose realism during hand motion tracking.

## Data Availability Statement

The original contributions presented in the study are included in the article and further inquiries can be directed to the corresponding author.

## Author Contributions

JI is the primary author who coded the SSC-CNN and performed the tests using the HANDS2017 and MSRA datasets. JI wrote the article with guidance from MM and BR. JI supervised the full research flow from concept discussion to code implementation. All authors contributed to the article and approved the submitted version.

## Conflict of Interest

The authors declare that the research was conducted in the absence of any commercial or financial relationships that could be construed as a potential conflict of interest.

## Publisher's Note

All claims expressed in this article are solely those of the authors and do not necessarily represent those of their affiliated organizations, or those of the publisher, the editors and the reviewers. Any product that may be evaluated in this article, or claim that may be made by its manufacturer, is not guaranteed or endorsed by the publisher.

## Footnotes

^1^ https://github.com/RBC-DSAI-IITM/SSCCNN

^2^ https://github.com/RBC-DSAI-IITM/SSCCNN

^3^https://tracklab.com.au/products/brands/ndi/ascension-trakstar/, Accessed March 2021.
